# Learning From Clinical Consensus Diagnosis in India to Facilitate Automatic Classification of Dementia: Machine Learning Study

**DOI:** 10.2196/27113

**Published:** 2021-05-10

**Authors:** Haomiao Jin, Sandy Chien, Erik Meijer, Pranali Khobragade, Jinkook Lee

**Affiliations:** 1 Center for Economic and Social Research University of Southern California Los Angeles, CA United States; 2 RAND Corporation Santa Monica, CA United States; 3 Department of Economics University of Southern California Los Angeles, CA United States

**Keywords:** dementia, Alzheimer disease, machine learning, artificial intelligence, diagnosis, classification, India, model

## Abstract

**Background:**

The Harmonized Diagnostic Assessment of Dementia for the Longitudinal Aging Study in India (LASI-DAD) is the first and only nationally representative study on late-life cognition and dementia in India (n=4096). LASI-DAD obtained clinical consensus diagnosis of dementia for a subsample of 2528 respondents.

**Objective:**

This study develops a machine learning model that uses data from the clinical consensus diagnosis in LASI-DAD to support the classification of dementia status.

**Methods:**

Clinicians were presented with the extensive data collected from LASI-DAD, including sociodemographic information and health history of respondents, results from the screening tests of cognitive status, and information obtained from informant interviews. Based on the Clinical Dementia Rating (CDR) and using an online platform, clinicians individually evaluated each case and then reached a consensus diagnosis. A 2-step procedure was implemented to train several candidate machine learning models, which were evaluated using a separate test set for predictive accuracy measurement, including the area under receiver operating curve (AUROC), accuracy, sensitivity, specificity, precision, F1 score, and kappa statistic. The ultimate model was selected based on overall agreement as measured by kappa. We further examined the overall accuracy and agreement with the final consensus diagnoses between the selected machine learning model and individual clinicians who participated in the clinical consensus diagnostic process. Finally, we applied the selected model to a subgroup of LASI-DAD participants for whom the clinical consensus diagnosis was not obtained to predict their dementia status.

**Results:**

Among the 2528 individuals who received clinical consensus diagnosis, 192 (6.7% after adjusting for sampling weight) were diagnosed with dementia. All candidate machine learning models achieved outstanding discriminative ability, as indicated by AUROC >.90, and had similar accuracy and specificity (both around 0.95). The support vector machine model outperformed other models with the highest sensitivity (0.81), F1 score (0.72), and kappa (.70, indicating substantial agreement) and the second highest precision (0.65). As a result, the support vector machine was selected as the ultimate model. Further examination revealed that overall accuracy and agreement were similar between the selected model and individual clinicians. Application of the prediction model on 1568 individuals without clinical consensus diagnosis classified 127 individuals as living with dementia. After applying sampling weight, we can estimate the prevalence of dementia in the population as 7.4%.

**Conclusions:**

The selected machine learning model has outstanding discriminative ability and substantial agreement with a clinical consensus diagnosis of dementia. The model can serve as a computer model of the clinical knowledge and experience encoded in the clinical consensus diagnostic process and has many potential applications, including predicting missed dementia diagnoses and serving as a clinical decision support tool or virtual rater to assist diagnosis of dementia.

## Introduction

The World Health Organization estimates that the number of people living with dementia worldwide is approximately 50 million and will almost triple by 2050 [1], with nearly 60% living in low- and middle-income countries like India [2]. Developing effective population-based interventions to address the rising burden of dementia depends on high-quality nationally representative data, which is often scarce in low- and middle-income countries. The Alzheimer’s and Related Disorders Society of India estimates that more than 3.7 million Indians have dementia. However, this figure is based on a meta-analysis of prevalence studies with estimated prevalence rates ranging from 0.6% to 10.6% in rural areas and from 0.9% to 7.5% in urban areas [3,4]. The high heterogeneity in reported prevalence could be due to a variety of methodological issues including regional variations and different diagnostic criteria [3].

The Longitudinal Aging Study in India (LASI) is the first and only nationally representative survey of the physical and cognitive health, economic welfare, and social well-being for the country’s aging population, with a sample of more than 70,000 individuals aged 45 years and older [3]. The Harmonized Diagnostic Assessment of Dementia for the Longitudinal Aging Study in India (LASI-DAD) further extends the LASI’s cognitive data collection by conducting in-depth neuropsychological tests and informant interviews for a subsample of the LASI respondents aged 60 years and older [3]. The design of the LASI-DAD closely follows the Harmonized Cognitive Assessment Protocol (HCAP), which was developed for the assessment of dementia and mild cognitive impairment in the US Health and Retirement Study (HRS) and its associated studies around the world to enable international research collaboration [5].

For conditions such as Alzheimer disease, dementia, and mild cognitive impairment, there is no single definitive diagnostic test. Hence, many clinical researchers rely on a clinical consensus diagnostic process, consisting of data review, adjudication, and consensus by a panel of expert clinicians [6,7]. However, for large population surveys, the gold standard of clinician in-person assessment of respondents and all relevant information from their informants and in-person consensus conference is costly [6,7]. One way to reduce the cost is to replace the in-person consensus conference with a web-based consensus diagnosis approach. This web-based method was implemented first in the Monongahela-Youghiogheny Healthy Aging Team Project [8] and then in the LASI-DAD [9], which developed an online clinical consensus diagnosis platform that provided the detailed information necessary for a clinical assessment [9] and obtained the Clinical Dementia Rating (CDR) for a subsample of the LASI-DAD participants (n=2528).

The objective of this study is to develop a machine learning model that uses information from the clinical consensus diagnosis in the LASI-DAD for classification of dementia. The resulting machine learning model can serve as a computer model of the clinical knowledge and experience encoded in the clinical consensus diagnostic process. Furthermore, the machine learning model can assist in predicting the dementia status of a subgroup of the LASI-DAD respondents who participate in the extensive cognitive tests and informant interviews but do not obtain the clinical consensus diagnosis due to missing information. The predicted data will become publicly available as a part of the LASI-DAD dataset for potential use in future studies.

This study is, to the best of our knowledge, the first machine learning study on dementia using a nationally representative sample from India. As a part of the LASI-DAD project, this study contributes to a global HCAP-based initiative to advance aging research based on the collection, sharing, and analysis of population data on cognition and dementia [10,11].

## Methods

### Overall Design

The LASI-DAD data were collected from the larger LASI project between October 2017 and March 2020 and involved a stratified random sample of 4096 individuals aged 60 years and over [3]. All LASI-DAD participants received an extensive cognitive assessment, and interviews were conducted with informants who knew the individual well. The collected data were used in clinical consensus diagnoses by a clinical expert panel to evaluate dementia status based on the CDR. A total of 2528 LASI-DAD participants received clinical consensus diagnoses, while the remaining 1568 individuals did not progress through the diagnostic process. This study developed a machine learning model using the same predictors as the LASI-DAD assessment and informant interview data in the clinical consensus diagnosis. The developed model predicts dementia diagnosis for individuals without consensus diagnosis.

### Assessment

The LASI-DAD protocol included a cognitive assessment; self-reported functional difficulties, depression, and anxiety; and an interview of an informant (a relative or friend who knows the individual well) about the respondent’s cognitive status and everyday activities. The main LASI collected rich data on sociodemographic status and health history, which were provided to clinicians for evaluation of the CDR. The data presented for the clinical consensus diagnoses were used as predictors in developing the machine learning model.

#### Cognitive Assessment

The Hindi Mental State Examination [12,13] is an assessment with questions related to tasks, including time orientation, place orientation, 3-word recall, and object naming. Example questions are “What is the year?” and “Can you tell me where we are now? What state? What city?” A summary score is calculated by summing the number of correct answers and ranges from 0 to 30, with a larger number indicating more correct answers.

The Telephone Interview for Cognitive Status (TICS) [14] is a widely used brief questionnaire with 3 questions. An example question is “What do people usually use to cut paper?” (Correct answer: scissors or shears.) The summary score is the total number of correct answers.

The Community Screening Instrument for Dementia (CSID) [15] is a brief assessment with 4 items, including “Where is the local market/store?” and “Point to the window and then the door.” The summary score is the total number of correct answers.

The judgment and problem-solving assessment [16] includes 5 questions, including “What is the difference between a lie and a mistake?” and “What will you do if you find a lost child on the road?” The summary score is the total number of correct answers.

Finally, there are 5 numeracy questions [17], with examples like “How many 25 paisa coins will you give me for one Rupee?” and “If 5 people all have the winning numbers in the lottery and the prize is 1000 Rupees, how much will each of them get?” The summary score is the total number of correct answers.

#### Self-Reported Functional Difficulties

Activities of daily living (ADLs) [18] assess difficulties in basic self-care tasks including dressing, walking, bathing, eating, getting in or out of bed, and using the toilet. Respondents can choose between yes and no when answering. The summary score is the total number of difficulties, with a higher score indicating more difficulties.

Instrumental activities of daily living [18,19] assess difficulties in daily-life tasks including preparing a meal, shopping for groceries, making phone calls, taking medications, doing housework, managing finances, and getting around or finding an address in an unfamiliar place. The summary score is the total number of difficulties, with a higher score indicating more difficulties.

The LASI mobility module assesses difficulties in 9 tasks such as walking 100 yards, sitting for 2 hours or more, and getting up from a chair after sitting for a long period. The summary score is the total number of difficulties, with a higher score indicating more difficulties.

#### Depression and Anxiety

Depression was assessed using the 10-item Center for Epidemiological Studies Depression Scale [20], which assesses 10 depressive symptoms in the past week such as having trouble concentrating, feeling depressed, and feeling tired or low in energy. The respondents can choose answers from rarely or never, sometimes, often, or most or all of the time, and scores are coded from 0 to 3. The summary score is calculated by summing the scores of each item and has a range from 0 to 30, with higher scores indicating more depressive symptoms.

Anxiety was assessed using a 5-item scale that is a subset of the Beck Anxiety Inventory [21], which measures anxiety symptoms in the past week, including a fear of the worst happening, being nervous, feeling hands tremble, a fear of dying, and feeling faint. The respondents can choose answers from never, hardly ever, some of the time, or most or all of the time, and scores are coded from 0 to 3. The summary score is calculated by summing the scores for each item and has a range from 0 to 15, with higher scores indicating more anxiety symptoms.

#### Informant Interview

The LASI-DAD asked respondents to nominate a close family member or friend as an informant who knows the respondent well, interacts with the respondent frequently, knows the respondent’s daily functions, and can report on the respondent [3]. The informant interview consisted of questions about the respondent’s functional status, social engagement, and memory.

The Informant Questionnaire on Cognitive Decline in the Elderly [22] includes 16 items asking the informant to compare the functional status and memory of the respondent to 10 years ago. Example questions include “How is the respondent at remembering things about family and friends, such as occupations, birthdays, and addresses compared with 10 years ago?” and “How is the respondent at handling money for shopping compared with 10 years ago?” The respondent can choose from much improved, a bit better, not much change, a bit worse, or much worse, and scores are coded from 1 to 5. The summary score is calculated as the average of all item scores.

The Blessed Dementia Scale [23] includes 8 questions for the informant to assess the change in performance and habits of the respondent. An example question is “How well is the respondent able to perform household tasks?” The informant can choose answers from no loss, some loss, and severe loss. If the informant answered some loss or severe loss, a further question is asked: “Is this loss due to physical reasons, mental reasons, or both?” The summary score is calculated by assigning 0 for no loss or loss only due to physical reasons, 0.5 for some loss attributed to mental reasons or both, and 1 for severe loss attributed to mental reasons or both and summing these scores, resulting in a summary score ranging from 0 to 8, with values in multiples of 0.5. There are 3 questions about the habits of the respondent. An example question is “Regarding eating, would you say the respondent feeds himself/herself without assistance, with minor assistance, with much assistance, or has to be fed?” The answer is scored from 1 to 4, with higher scores indicating more difficulties. The summary score is the average score of the 3 items.

Additionally, there are questions for the informant to assess the signs of cognitive change, signs of cognitive impairment, and everyday activities. Example questions include “Does the respondent have difficulty in adjusting to change in the respondent’s daily routine?” for assessing signs of cognitive change, “Has there been a general decline in the respondent’s mental functioning?” for assessing signs of cognitive impairment, and “How often does the respondent go to work or volunteer?” for assessing everyday activities.

#### Sociodemographic Variables

The sociodemographic variables include age, marital status (married or not), gender, and years of education.

#### Health History

Health history includes systolic and diastolic blood pressure and previous diagnosis of stroke, heart disease, diabetes, hypertension, depression, dementia, psychiatric problems, neurological problems, vision impairment, and hearing impairment.

### Clinical Consensus Diagnosis of Dementia

Obtaining the ground truth is a challenge for all machine learning studies on dementia because there is no single definitive test of the disease. For the basis of the clinical diagnosis of dementia, clinicians used the CDR [16], a global rating device first introduced in a prospective study of patients with dementia [24] that is now widely used to measure dementia severity [25,26]. The CDR comprises 6 cognitive and functional domains [16]: (1) memory, (2) orientation, (3) judgment and problem solving, (4) community affairs, (5) home and hobbies, and (6) personal care. Clinicians complete the CDR ratings based on cognitive test results and informant reports. As noted earlier, the LASI-DAD project built a web-based approach to reach diagnostic consensus [3]. For each individual, at least 3 clinicians were assigned to the first round of review. Each clinician reviewed the case and provided ratings for the subdomains, and based on the subdomain ratings, the CDR algorithm automatically generated a global rating: 0 (normal), 0.5 (very mild dementia), 1 (mild dementia), 2 (moderate dementia), or 3 (severe dementia). For cases where individual global ratings differed, an automatic email was sent to the assigned reviewers, giving them a chance to review the case and read other raters’ comments and update their ratings, if desired [3]. This second round of review might reach a consensus for additional cases. For cases where consensus was not reached after the second round of review, a group of clinicians discussed the case through a virtual consensus meeting to determine the global CDR rating. This clinical consensus diagnostic process can surpass the accuracy of individual expert diagnoses and is considered the gold standard for clinical diagnosis of dementia [27]. An individual was classified as having dementia if the global CDR rating from the clinical consensus diagnostic process was equal to or greater than 1.

### Statistical Analysis

This study generated descriptive statistics of the data for developing the machine learning model. Available data were then divided into a training set with a random selection of 70% of the sample and a test set involving the remaining 30% of the sample. We trained several candidate machine learning models using the training set, including stochastic gradient boosting, random forest, support vector machine, elastic net, multivariate adaptive regression splines, and multilayer perceptron. Stochastic gradient boosting is an ensemble learning method that produces a prediction model based on weak prediction models, typically decision trees [28]. Random forest constructs a multitude of decision trees at model training and outputs the ultimate prediction that is the mode of the individual trees [29]. Support vector machine constructs hyperplanes to separate different categories of training samples [30]. A radial basis function kernel was used in this study with a support vector machine to construct nonlinear separations [30]. Elastic net is a regularized regression method that linearly combines the L1 and L2 regularization to achieve improved predictive accuracy [31]. The multivariate adaptive regression splines model is a nonparametric regression technique that automatically models nonlinear relationships [32]. Finally, multilayer perceptron is a type of fully connected artificial neural networks with an input layer, one or more hidden layers, and an output layer [30]. A multilayer perceptron with multiple hidden layers is often known as a type of deep neural network [33]. The model training process as described below tuned the number of hidden layers, number of neurons in each layer, and a weight decay parameter for reducing model overfitting to select the best structure of the neural network.

We trained the candidate machine learning models using a 2-step process. First, we fitted the models based on the training set using repeated cross-validation with 10 repetitions and 10 folds of validation [34]. The objective of this step was to optimize the models’ overall discriminative abilities by tuning model meta-parameters, such as the number of decision trees in a random forest and the number of hidden layers in a multilayer perceptron, and using the fitted models to generate predicted risk scores for each training sample (calculated as 100 times the predicted probability of dementia). Whenever possible, a weight inversely proportional to the number of individuals with dementia in the training set was used to account for the imbalance between individuals with versus individuals without dementia in the data. The overall discriminative ability was evaluated by the area under the receiver operating curve (AUROC), which is a measure based on the sensitivity (ie, number of true positive divided by all positive cases) and specificity (ie, number of true negative divided by all negative cases) of different cutoffs for the predicted risk scores. AUROC has a range from 0 to 1, and an AUROC score of more than 0.9 is considered outstanding [35]. In the second step, we trained a majority-voting process that outputted the final classification of dementia by combining 4 weak classifications derived from the predicted risk score. For each individual, the process attached 4 respective group memberships based on the individual’s depression and anxiety assessment scores (in the top quartile or not) and whether the individual had vision or hearing impairment. The cutoff scores for each group were selected to maximize the F1 score, which is a summary score calculated from sensitivity and precision (true positive cases divided by the total number of predicted positive cases). Based on the comparisons between the predicted risk score and group-specific cutoffs, each individual received 4 respective weak classifications. An individual was assigned as having dementia if at least 3 weak classifications were positive. Design of this majority-voting process was informed by the clinical evidence that cognitive decline and daily life difficulties may be attributed to alternative conditions other than dementia, such as depression, anxiety, and vision and hearing impairment [36-40]. The above described model training process was implemented by using the R functions trainControl and train in the caret package [41].

We tested the predictive accuracy of the candidate machine learning models using the test set. Predictive accuracy was evaluated by the AUROC of the predicted risk scores and accuracy, sensitivity, specificity, precision, F1 score, and kappa of the final classifications. We selected the model with the highest kappa as the ultimate prediction model because kappa measures the overall agreement between the predicted classifications and the clinical consensus diagnoses and corrects for the imbalance between positive and negative cases. In general, kappa between .60 and .80 indicates substantial agreement and kappa greater than .80 indicates almost perfect agreement [42]. We further compared the overall accuracy and agreement with the final consensus diagnoses between the selected machine learning model and clinicians who participated in the clinical consensus diagnostic process. Finally, we applied the selected model to predict dementia for individuals without clinical consensus diagnoses. As mentioned earlier, the predicted data will be publicly available as a part of the LASI-DAD dataset.

## Results

The sample used to develop the machine learning model included 2528 individuals from the LASI-DAD who received a clinical consensus diagnosis of dementia (Table 1). The sample included 192 individuals living with dementia.

Random selection split the data into a training set with 1770 individuals and a test set with 758 individuals. There were 138 individuals diagnosed with dementia in the training set and 54 individuals diagnosed with dementia in the test set. Evaluation results of the candidate machine learning models on the test set are shown in Table 2. The tuned multilayer perceptron has one hidden layer with 5 neurons. All candidate models achieved outstanding discriminative ability with AUROC >.90 and similar accuracy and specificity. However, the support vector machine outperformed the other models with the highest sensitivity, F1 score, and kappa and the second highest precision. The support vector machine was selected as the ultimate model since it has the best kappa, indicating the best overall agreement between predicted classifications and clinical consensus diagnoses.

Further examination revealed that accuracy of the selected prediction model (ie, support vector machine) is similar to that of the clinicians who participated in the clinical consensus diagnosis. A total of 12 clinicians participated in the consensus diagnostic process for the 758 individuals in the test set. Compared with the final consensus diagnoses, the average accuracy of clinicians was 0.96 (95% CI 0.94-0.98) and the average kappa was .75 (95% CI 0.61-0.88). There were no significant differences between the selected prediction model and the participating clinicians (accuracy *P*=.64 and kappa *P*=.46).

Application of the selected prediction model to the 1586 individuals without clinical consensus diagnoses results in 127 individuals classified as living with dementia. Hence, the unweighted estimated dementia prevalence in the total sample is (127+192)/4096=7.8%. Applying sampling weights, we can estimate the prevalence in the population as 7.4%.

**Table 1:** Descriptive statistics of the data used to develop the machine learning model (n=2528).

Characteristics		Unweighted group	Weighted group
Age (years, range 6-103), mean (SD)		68.69 (7.50)	68.54 (7.35)
Married, n (%)		1672 (66.14)	1856 (67.10)
Female, n (%)		1204 (47.63)	1403 (50.70)
Education (years, range 0-20), mean (SD)		3.64 (4.60)	3.40 (4.57)
Blood pressure (systolic, range 76.5-225.0), mean (SD)		138.38 (23.35)	138.06 (23.62)
Blood pressure (diastolic, range 47.5-137.0), mean (SD)		82.73 (12.46)	82.73 (12.46)
**Previous diagnosis, n (%)**			
	Stroke	82 (3.24)	88 (3.18)
	Heart disease	155 (6.13)	146 (5.28)
	Diabetes	376 (14.87)	352 (12.72)
	Hypertension	964 (38.13)	939 (34.12)
	Depression	21 (0.83)	23 (0.83)
	Dementia	26 (1.03)	25 (0.90)
	Psychiatric problems	17 (0.67)	14 (0.51)
	Neurologic problems	58 (2.29)	63 (2.28)
	Vision impairment	1113 (44.03)	1222 (44.16)
	Hearing impairment	712 (28.16)	770 (27.83)
HMSE^a^ (range 0-30), mean (SD)		22.11 (5.87)	22.15 (5.73)
TICS^b^ (range 0-3), mean (SD)		2.01 (0.92)	1.97 (0.93)
CSID^c^ (range 0-4), mean (SD)		3.31 (0.94)	3.29 (0.95)
Judgment and problem solving (range 0-5), mean (SD)		2.21 (1.51)	2.14 (1.52)
Numeracy (range 0-9), mean (SD)		3.85 (2.64)	3.83 (2.61)
ADL^d^ (range 0-6), mean (SD)		1.34 (1.76)	1.30 (1.75)
IADL^e^ (range 0-7), mean (SD)		2.27 (2.24)	2.27 (2.23)
Difficulties in mobility (range 0-9), mean (SD)		3.72 (2.92)	3.65 (2.94)
Depressive symptoms (range 0-30), mean (SD)		9.92 (5.25)	10.30 (5.17)
Anxiety symptoms (range 0-15), mean (SD)		2.94 (3.30)	3.09 (3.37)
IQCODE^f^ (range 1-5), mean (SD)		3.51 (0.56)	3.48 (0.54)
Blessed Dementia Scale–changes in habits (range 1-3.67), mean (SD)		1.09 (0.32)	1.07 (0.28)
Blessed Dementia Scale–changes in performance (range 0-8), mean (SD)		1.26 (1.71)	1.20 (1.63)
**Global CDR^g^, n (%)**			
	0 (no dementia)	768 (30.38)	854 (30.86)
	0.5 (very mild dementia)	1568 (62.03)	1726 (62.38)
	1 (mild dementia)	162 (6.41)	160 (5.78)
	2 (moderate dementia)	25 (0.99)	24 (0.87)
	3 (severe dementia)	5 (0.20)	2 (0.07)
Diagnosis of dementia (global CDR ≥1), n (%)		192 (7.59)	186 (6.72)

^a^HMSE: Hindi Mental State Examination.

^b^TICS: Telephone Interview for Cognitive Status.

^c^CSID: Community Screening Instrument for Dementia.

^d^ADL: activity of daily living.

^e^IADL: instrumental activity of daily living.

^f^IQCODE: Informant Questionnaire on Cognitive Decline in the Elderly.

^g^CDR: Clinical Dementia Rating.

**Table 2:** Predictive performance of candidate machine learning models based on evaluation on the test set.

Model	AUROC^a^	Accuracy	Sensitivity	Specificity	Precision	F1	Kappa
Stochastic gradient boosting	.94	.95	.67	.97	.64	.66	.63
Random forest	.95	.95	.67	.98	.68	.67	.65
Support vector machine	.95	.96	.81	.97	.65	.72	.70
Elastic net	.95	.94	.65	.96	.58	.61	.58
Multivariate adaptive regression splines	.94	.94	.69	.96	.60	.64	.61
Multilayer perceptron	.95	.95	.67	.97	.65	.66	.63

^a^AUROC: area under the receiver operating curve.

## Discussion

### Principal Findings

This study developed a machine learning model that uses clinical consensus diagnosis on dementia in a nationally representative survey of individuals aged 60 years and older from India. The ultimate prediction model is a support vector machine model with radial basis function kernel trained on a 2-step process. Validation results suggest that the prediction model has outstanding discriminative ability (AUROC >.90) and substantial agreement with clinical consensus diagnosis (kappa between .60 and .80). Compared with clinicians who participated in the clinical diagnostic process, the machine learning model demonstrates similar overall accuracy and agreement with the final consensus diagnoses. This finding suggests that the prediction model may serve as a decision support tool or even a virtual participating rater in the clinical consensus diagnostic process.

The developed machine learning model has many potential applications. First, as shown in this study, the model can be used to predict dementia diagnosis for individuals without clinical consensus diagnosis in the LASI-DAD project. Future data users may use the predicted data for various purposes, such as estimating the prevalence of dementia in India or examining risk factors of dementia. Second, the prediction model can be built into the online consensus website as a clinical decision support tool or a virtual participating rater to replace one of the clinicians. The next wave of LASI-DAD data collection will implement the machine learning model developed in this paper as a participating virtual rater to replace one of the clinicians in the consensus diagnostic process for a proportion of cases. The implementation data will be used to evaluate whether using the model would impact the accuracy and efficiency of the consensus diagnostic process. Using the machine learning model to replace one of the clinicians has the potential to further reduce the cost associated with implementing the clinical consensus diagnosis while maintaining expert clinicians as the dominating force in the diagnostic process. That is, at least 2 clinicians will still be included in the diagnostic process and any inconsistency between the human and virtual raters will be resolved through the standard consensus process, which involves the meeting of a group of expert clinicians to discuss cases. Third, since the design of the LASI-DAD closely follows the HCAP to facilitate international collaboration, the developed model may be used in other HCAP-based studies as an external classification tool for dementia in the absence of clinical ratings for those studies. Fourth, the developed machine learning model can serve to capture the significant clinical knowledge and experience encoded in the clinical consensus diagnostic process. Further examination of the computer model using meta-modeling techniques [43] may generate in-depth understanding of the clinical consensus diagnostic process, such as identifying the top influential assessments for making a diagnosis of dementia. Fifth, since there is no definitive diagnostic test of dementia, tracking the misclassified cases by the machine learning model in the next few years may reveal whether those cases are actual false classifications. Finally, the future waves of LASI-DAD data will be used to identify potential improvements and provide further validation of the current model to ensure its predictive accuracy in the long term.

Another important finding of the study is that the 2-step training process may outperform the standard single-step training process for the classification problem of dementia. Our analysis shows that adding the majority-voting as a second step to the training process reduces approximately one-fourth of misclassifications on the test set. The kappa of the model derived from the 2-step training procedure also outperformed the kappa of the model derived from a standard, repeated cross-validation–based training process that directly optimizes the kappa. An important observation of models derived from the standard training process is that these models tend to overfit the training set, as evidenced by the accuracy (>99%) on the training set for most models. Adding the majority-voting process as a second step reduces overfitting, thereby improving predictive performance on the test set.

### Comparison With Prior Literature

The techniques of machine learning have been applied to the examination of survey data for predicting a variety of diseases, such as anxiety [44-46], depression [44,46-51], and dementia [52-54]. This study is based on a nationally representative survey involving a clinical consensus diagnosis of dementia. The number of comparable datasets is limited. The Aging, Demographics, and Memory Study (ADAMS) includes clinical consensus diagnoses for a subsample of 856 individuals aged 70 years and older in the United States from the HRS [55]. Another similar nationally representative dataset is the Hellenic Longitudinal Investigation of Aging and Diet (HELIAD) study with a sample of 1050 individuals aged 65 years and older in Greece [56]. The LASI-DAD dataset used in this study expands the clinical consensus diagnosis to a larger sample with a broader age range than the ADAMS and HELIAD.

Due to the limited number of available nationally representative datasets with a clinical diagnosis of dementia, only a few machine learning studies have used such type of data. Hurd et al [57] developed an ordered probit model to predict the probability of dementia using the ADAMS dataset. The predictive performance of the model is unclear since validation results based on a randomly selected test set were not reported. Nevertheless, such predicted probability of dementia was used in a subsequent machine learning study by de Langavant et al [53] to test the relevance of an unsupervised learning model based on the larger HRS data (HRS is the parent study of ADAMS). de Langavant et al [54] developed a similar unsupervised learning model to assist the estimation of dementia prevalence in 10 nationally representative surveys. Na [52] developed a supervised machine learning model using data from the Korean Longitudinal Study of Aging to facilitate automatic classification of dementia. However, dementia in this paper was classified by the Mini-Mental State Examination scores below one standard deviation of the mean scores of age by educational level stratified groups. Since screening results can misclassify dementia [58], such classification can only serve as a weaker ground truth of dementia than clinical consensus diagnosis.

The majority of existing machine learning studies on dementia are based on neuroimaging data (see systematic review like Pellegrini et al [59]). In contrast, our study relies on cognitive tests and informant reports, which are easier to obtain for a large sample than neuroimaging data. These tests and questionnaires have been carefully selected in a rigorous process developing the multicountry HCAP [5,60] and translated and adapted to fit the Indian context [61]. The psychometric properties of the tests in the LASI-DAD sample have been assessed favorably [62]. However, the measures are not perfect. Specifically, the literature has found that informant reports of individuals’ limitations and cognitive decline, while highly correlated with other measures, may differ from the individuals’ reports and reports from health professionals, with the discrepancy systematically depending on the type of proxy (eg, whether caregiver or not) [63-66]. Predictive accuracy as assessed by commonly used measures like overall accuracy, AUROC, and kappa of the model developed in this paper is comparable to or, in some cases, outperforms the neuroimaging-based machine learning models. However, since the survey and neuroimaging data are different and there is no definitive diagnosis of dementia, we caution the use of such direct comparison as a criterion to judge the predictive performance of a model.

### Limitations

This study has several limitations. Although the dataset for developing the machine learning model more than doubles the size of similar nationally representative datasets involving a clinical consensus diagnosis of dementia and is much larger than many clinical datasets, the sample size is still limited from a big data perspective. This is evident from the results that the multilayer perceptron with one hidden layer and other so-called shadow learning models like the support vector machine outperform the deep neural network model with multiple hidden layers in this paper. Typically, deep learning techniques outperform shadow learning techniques when the sample size is very large [67]. Second, as mentioned above, informant reports may to some extent systematically vary with the informant type, and neuroimaging data may give a more accurate assessment of the individual than our combination of cognitive tests and informant reports. However, as described above, all measurements used in this study are well validated and widely used worldwide in population-based aging studies. Third, the number of clinicians participating in the diagnostic process for test set samples is 12, which may not be large enough to be representative. Fourth, although the predictive accuracy of the support vector machine model is high, it is difficult to directly interpret the model for generating in-depth understanding of the dementia diagnostic process. As mentioned above, meta-modeling techniques may be useful for improving the interpretability of the model [43]. Finally, even though clinical consensus diagnosis is considered the gold standard in diagnosing dementia [27], it is not without errors. Tracking the misclassified cases in the next few years may reveal whether misclassifications made by the machine learning model are actually false classifications.

Further research will be needed to assess whether the model can be used for individuals from other countries. Comparable data (cognitive tests, informant reports, and online clinical consensus ratings) will be available in the near future for at least 2 other countries (the United States and South Africa, and possibly China later on), which will allow such assessments. An ongoing global initiative known as the Gateway to Global Aging Data is actively working on creating a harmonized multinational dataset, with the LASI-DAD project a part of the initiative [10]. Even if the model developed in this paper does not readily generalize to data from another country without adaptation, the 2-step training process developed in this paper may still be useful for developing similar machine learning models for dementia. In addition, the current model may be recalibrated for a similar population-based dataset from another country by including the predicted risk score or dementia status from the current model as one of the candidate predictors for training a dedicated model for that country [68]. Further improvements in the model may be possible when data from the second wave of LASI-DAD study become available, including online consensus ratings by clinicians who will be able to evaluate data from 2 observations spaced approximately 4 years apart.

### Conclusion

This study develops a machine learning model that learns from clinical consensus diagnoses of dementia from a nationally representative survey of the aging population in India to facilitate the automatic classification of dementia. The developed model has outstanding discriminative ability and substantial agreement with clinical consensus diagnoses of dementia. The model can serve as a computer model of the clinical knowledge and experience encoded in the clinical consensus diagnostic process and has many current and potential applications, including prediction for missing dementia diagnoses and serving as a clinical decision support tool to assist diagnoses of dementia. The predicted missing dementia diagnoses will be released as a part of the LASI-DAD data for future use in broader aging research. The LASI-DAD study also plans to implement and test the developed model as a participating virtual rater in the consensus diagnostic process in the next wave of data collection. The future implementation data will be valuable for identifying potential further improvements of the model and ensuring its predictive accuracy in the long term.

## Abbreviations

ADAMS: Aging, Demographics, and Memory Study
ADL: activity of daily living
AUROC: area under receiver operating curve
CDR: Clinical Dementia Rating
CSID: Community Screening Instrument for Dementia
HCAP: Harmonized Cognitive Assessment Protocol
HELIAD: Hellenic Longitudinal Investigation of Aging and Diet
HRS: Health and Retirement Study
LASI: Longitudinal Aging Study in India
LASI-DAD: Harmonized Diagnostic Assessment of Dementia for the Longitudinal Aging Study in India
TICS: Telephone Interview for Cognitive Status
